# Innovation in Alternative Food Sources: A Review of a Technological State-of-the-Art of Insects in Food Products

**DOI:** 10.3390/foods11233792

**Published:** 2022-11-24

**Authors:** Pedro Paulo Lordelo Guimarães Tavares, Matheus dos Santos Lima, Luiggi Cavalcanti Pessôa, Roberta Barreto de Andrade Bulos, Thâmilla Thalline Batista de Oliveira, Larissa Farias da Silva Cruz, Denilson de Jesus Assis, Elba Santos da Boa Morte, Cláudio Vaz Di Mambro Ribeiro, Carolina Oliveira de Souza

**Affiliations:** 1 Graduate Program in Food Science (PGALI), Faculty of Pharmacy, Federal University of Bahia, Salvador 40170-115, Bahia, Brazil; 2 Undergraduate Program in Pharmacy, Federal University of Bahia, Salvador 40170-115, Bahia, Brazil; 3 Graduate Program in Chemical Engineering (PPEQ), Polytechnic School, Federal University of Bahia, Salvador 40210-630, Bahia, Brazil; 4 Environment Department, Senai Cimatec University Center, Salvador 41650-010, Bahia, Brazil; 5 School of Exact and Technological Sciences, Salvador University, Salvador 41820-021, Bahia, Brazil; 6 Graduate Program in Food, Nutrition and Health (PPGANS), School of Nutrition, Federal University of Bahia, Salvador 40110-907, Bahia, Brazil; 7 School of Veterinary Medicine and Animal Science, Federal University of Bahia, Salvador 40170-110, Bahia, Brazil; 8 Department of Bromatological Analysis, College of Pharmacy, Federal University of Bahia, Salvador 40170-115, Bahia, Brazil

**Keywords:** patent study, market trend, edible insect, food industry, prospection, novelty

## Abstract

Insects present great potential for the food industry due to their easier rearing conditions and high nutritional value, in comparison with traditional livestock. However, there is a lack of evaluation of the technological status of food products developed with edible insects. Therefore, this study aims to analyze the emergent technological and scientific applications of edible insects in the food industry through a prospective study of patent documents and research articles. Espacenet was used as a research tool, applying the terms Insect, Pupa, Larva, or Nymph and the codes A23L33 and A23V2002. A total of 1139 documents were found—341 were related to the study. Orbit^®^ was used to evaluate technological domains and clusters of concepts. Scopus database research was performed to assess the prevalence of insect research, with the term “edible and insect*”. The main insects used were silkworms, bees, beetles, mealworms, crickets, and cicadas. Protein isolates were the predominant technology, as they function as an ingredient in food products or supplements. A diverse application possibility for insects was found due to their nutritional composition. The insect market is expected to increase significantly in the next years, representing an opportunity to develop novel high-quality/sustainable products.

## 1. Introduction

It is known that edible insects are an alternative to replace traditional food sources. There are several other potential benefits of its creation, such as environmental, economic, and social [1]. The concept of biorefinery, for example, deals with the conversion of biomass from agroindustrial waste into various products with higher added value [2,3]. An insect biorefinery corresponds to an integrated technology that allows the valorization of organic waste through its transformation into biomass rich in nutrients and bioactive compounds [4].

World hunger, sustainable food production, and waste reduction are global challenges for current and future generations. It is estimated that approximately one-third of the food produced in the world is lost [5]. In a conjunction report by FAO; IFAD; UNICEF; WFP; WHO [6], from 702 to 828 million people were suffering from hunger in 2021. Moreover, the COVID-19 pandemic resulted in a growing number of malnutrition by around 150 million since its beginning up until recent days [6]. Another recent and aggravating situation refers to the conflict between Russia and Ukraine, relevant countries regarding the exportation of agricultural products. With the ceasing of Ukrainian exports, commodities have highly increased in price worldwide and future harvests may not compensate for this issue. This may incur an increase in food insecurity by about 47 million people, compared with a context without war [7]. Therefore, malnutrition has been growing considerably in the last few years, which indicates a situation of urgency that needs to be reversed. Research in the innovation area has focused on producing alternative sources that can combine efforts aimed at food and nutrition security [8,9]. Considering that foods of animal origin generally have protein quality superior to plants [10], insects are presented as potential ingredients for the food industry [8].

Edible insects are considered excellent sources of proteins, lipids, minerals, and vitamins [11,12,13]. Moreover, they contain antioxidant compounds [14], essential amino acids [15], and polyunsaturated fatty acids [16]. Insects can be consumed whole or used as ingredients to fortify food formulations [17], fulfilling nutritional needs in countries with a high prevalence of malnutrition and food insecurity [17]. Furthermore, they can serve as nutritious food for healthy populations [18], using a source whose environmental impact is lower compared to conventional animal proteins, responsible for great environmental impacts, such as high land and water usage, and greenhouse gas emissions produced by enteric fermentation and manure storage [19,20].

Studies indicate an increase in interest regarding the use of insects as alternative ingredients in the preparation of traditional foods, such as bread [21,22,23], pasta [24], cereal bars [25], soup [26], biscuits [27], sausage [28], and extruded snacks [29]. The insertion of 10% flour/powder of the cinereous cockroach (*Nauphoeta cinerea*) in bread, increased protein intake by 12.90% [30]. Moraes et al. [31] when developing cereal bars with flour/powder of *Tenebrio molitor* larvae, observed significant increases in lipid content (from 20.39 to 30.58%) and total protein (from 13.68 to 21.08%) for the elaborated product.

Edible insects are already produced on a commercial scale in several countries around the world. Following the report encouraging the production and consumption of insects by FAO in 2013 [32], countries in North America, Europe, Africa, and Asia have installed insect production systems for human and animal consumption, called insect farms. These are structures that must meet specific requirements for proper operation, considering that each species has its intrinsic characteristics [33]. European agencies have published regulations for the production and consumption of edible insects as human food, such as the Federal Agency for the Safety of the Food Chain of Belgium (AFSCA), which released general standards for the rearing and marketing of insects and derivatives in 2014; and the marketing permission published by the Swiss Federal Food Safety and Veterinary Office (FSVO) in 2017, which now allows the marketing of products based on crickets, grasshoppers, and larvae of *Tenebrio molitor* [34,35]. The European Commission for Food Safety, through Regulation 2015/2283, authorized the use of *Tenebrio molitor* larvae in food for human consumption and guaranteed its safety [36].

The global edible insects market presented in 2018 a value close to USD 400 million, with a forecast to grow approximately three times by 2023, indicating great investment potential [37]. The mass production of edible insects, on the other hand, was predicted at approximately 200,000 tons in 2020 and should increase to 1.20 billion tons in 2025 [38,39,40]. Considering that this is a market with great potential, the development of a technological prospection is important to identify trends and potential investments in the area.

Patent documents represent an invention deposited in a specific technological field; therefore, as one of the main sources of information regarding new technologies [41]. The analysis of patent documents generates valuable information for organizations, by enabling the determination of the originality of their inventions, as well as the verification of technologies protected by competing organizations [40]. Moreover, it is an important tool in decision-making and can facilitate the appropriation with quality of Intellectual Property, by increasing the critical sense and broadening the vision of existing technologies and opportunities [42].

The present study, therefore, aims to evaluate the application of edible insects in the food industry through a prospective study, focusing on elements, such as insects most predominantly used in patents, main countries holding technologies, types of products developed, and claims/functions associated with these products.

## 2. Review Methodology

### 2.1. Patent Search

The methodology consisted of searching for patent documents in the international database Espacenet in August 2022. Espacenet is a database of the European Patent Office (EPO), where patent documents from more than 100 countries can be found. The search strategy used keywords to be found in the titles and/or abstracts and two codes directly associated with the search objective. The keywords (Insect, Pupa, Larva, or Nymph) were chosen to consider all stages of insect development and avoid underestimating the data. In addition, the codes A23L33 (Modifying nutritive qualities of foods; Dietetic products; Preparation or treatment thereof) and A23V2002 (Food compositions, function of food ingredients or processes for food or foodstuffs) were selected in order that the search would retrieve only documents directly associated with the food industry. The Boolean operators (OR) and (AND) were used in order that at least one code and one keyword were present in the documents evaluated. The truncation operator (*) was used to find derivations of the keywords. The documents selected for data processing were those that presented greater proximity to the proposed theme. The information regarding the available documents was exported to Microsoft 365 Excel using the CSVed software, for later analysis. The Orbit^®^ was used in association with Espacenet to evaluate technological domains and clusters of concepts more prevalent in patent documents. Orbit^®^ is a private platform developed by Questel company that provides users with patent research and analysis options.

The descriptors selected in the search for patent documents are highlighted in bold in Table 1. The search resulted in a total of 1139 patent documents. After reading the titles and abstracts, only 341 documents were within the proposed theme and were selected.

### 2.2. Scientific Documents Search

A search in the scientific database Scopus was performed to assess the prevalence of insect research until August 2022. The term “edible and insect*” was used, to be found in the titles, abstracts, or keywords of documents. The search resulted in a total of 2623 documents. Information, such as country/territory, subject area, and year of publication was assessed using Scopus website tools to compare with the results from the patent documents. Moreover, some of the main articles found were used in this review to provide a dialogue between patent documents and scientific research.

## 3. Annual Evolution in the Deposit of Patent Documents and Scientific Production

**Figure 1:**
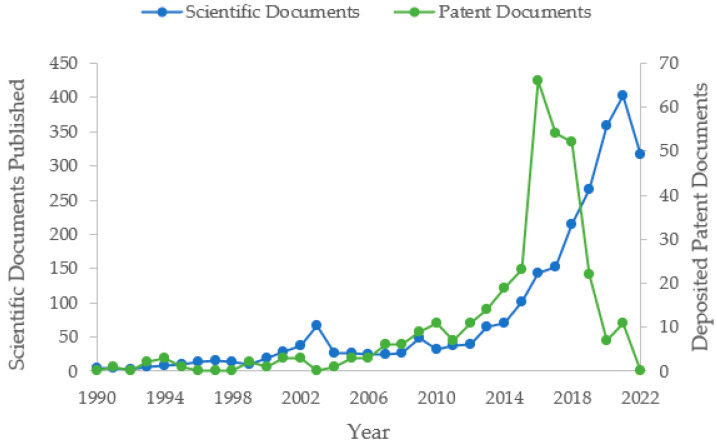
Global trend in the publication of patent documents and scientific papers * associated with edible insects. * Number of scientific documents published between 1990 and 2022 on edible insects, according to the Scopus database.

Figure 1 shows the deposit of patent documents and scientific production related to the development of food products containing edible insects. In 1991, the first patent registration occurred in China—CN1055644A [43]. This invention refers to the development of food with health claims that use cicadas as an ingredient.

In the 1990s, the number of patent document filings related to the evaluated technology was low. However, as of 2007, it is possible to observe a slight increase in the average number of documents filed. In 2010, functional foods with insects represented the majority of patented technologies (36.36%), followed by food supplements (27.27%). As an example of technologies developed in 2010, patent CN102370666A [44] refers to a powdered energetic supplement for athletes, obtained from freeze-dried water beetle (*Cybister tripunctatus orientalis*). Patent KR20110121223A [45] deals with a functional food powder, encapsulated, containing silkworms, with the ability to control the effects of menopause.

In 2010, the Food and Agriculture Organization (FAO) published the document entitled “Forest insects as food: humans bite back” [46], reporting the proceedings of an international workshop held in Chiang Mai, Thailand, in 2008. The workshop was developed by FAO Regional Office for Asia and the Pacific, which invited experts in the field of entomophagy to discuss topics, such as management, processing, marketing, and consumption of edible forest insects, in addition to assessing the economic potential of insect production by local farmers [46].

As of 2013, the technology showed an exponential increase in annual average deposits, a trend that may be associated with the dissemination of a document entitled “Edible insects: future prospects for food and feed security” by FAO [32]. This document is a collaborative production between FAO and the Entomology Laboratory of Wageningen University in the Netherlands and aimed to conduct a comprehensive assessment of the contributions of edible insects to food security and livelihoods in developed and developing countries, and stimulate the national and international agencies to invest in the scientific research in sustainability and food security [32]. Nevertheless, in 2013, the researcher Arnold van Huis published the review article “Potential of Insects as Food and Feed in Assuring Food Security” [1], indicating a trend in the use of edible insects as alternative nutritional sources of quality and sustainability, thus arousing the interest of the scientific community in conducting new research in the area.

The facts that occurred in the year 2013 may represent milestones responsible for the subsequent peak in the filing of documents that occurred in 2016 (66 patents filed). Most patents filed this year had their origin in Asia (97.40%), where 61.04% of the filings occurred in China and 36.36% occurred in the Republic of Korea, countries with high technological development and consolidated entomophagy culture [16,47].

The growth in the number of patent document filings has also been accompanied by an increase in the number of scientific production. Van Huis and Oonincx [18] demonstrated that the interest in insect research is illustrated by the many articles referenced. In 2010, there were 32 documents listed in the Scopus database using the term “edible and insect*”, in 2016 this number increased by over 346% (144), peaking in the years 2020 and 2021 with 361 and 402, respectively (query on 14 August 2022) (Figure 1). Finally, in the years 2020, 2021, and 2022, a reduction in the number of patent deposits is perceived. This may be associated with the secrecy period, which can last up to 18 months.

In general, there is a progressive increase in the number of patent documents and scientific articles, which indicates that the technology is still recent and has room for growth and development, not yet presenting scientific-technological maturity. Entomophagy is an ancient practice [48]; however, only after FAO’s efforts, it was noticed a stimulus in the production of technologies and research within the theme, which have been developed very recently. Some authors discuss the presence of several gaps to be solved by conducting more scientific studies, mainly focused on nutritional quality, management, cultivation, and ethical issues in insect production, which are still scarce [49,50].

## 4. Main Technology Domains

**Figure 2:**
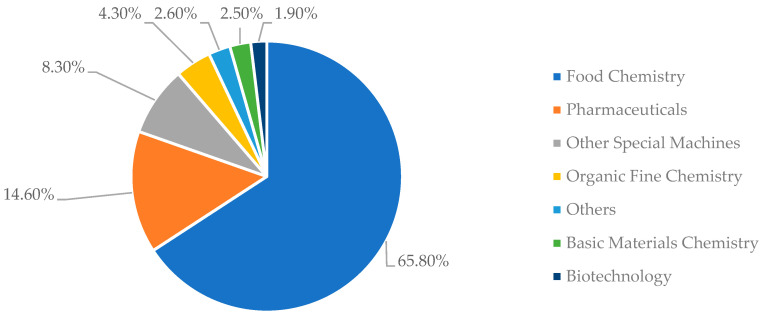
Technological domains present in patent documents based on the use of edible insects and food products. Source: Q. Orbit^®^.

Figure 2 deals with the technological domains pertaining to the patent documents selected in Orbit. Food chemistry presented the greatest contribution, with 65.80%, followed by the pharmaceutical area, with 14.60%. With regard to the areas of study present in the scientific documents evaluated in Scopus, there is a greater prevalence of Agricultural and Biological Sciences (42.08%) as well as Biochemistry, Genetics, and Molecular Biology (11.71%). The predominance of these sectors can be recognized by the fact that most patent documents and scientific studies are dedicated to the use of insects to develop nutritionally rich food products (emphasis on proteins and lipids) or as sources of bioactive compounds with various beneficial effects to the human body [51]. Considering that the use of insects in the food industry is a subject still in expansion, from the identification of technological domains less studied it is possible to verify opportunities for the development of new technologies [41]. In this case, the domains of Organic fine chemistry, Biotechnology, Other special machines, and Basic materials chemistry are highlighted as less explored areas. As examples of applications of these domains: The document CN111000021A [52] deals with equipment capable of producing insect protein powder for human consumption. It is composed of the main base, feeding device, dryer, crusher, supercritical extraction machine, and freeze-dryer. The document KR102098079B1 [53] deals with a fermentation process of *Protaetia brevitarsis* by *Bacillus subtilis* to develop a digestible food formula with medicinal properties associated with liver function improvement.

## 5. Main Depositors

**Figure 3:**
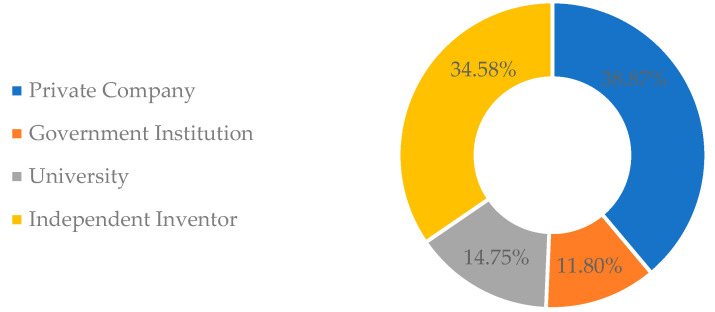
Distribution of applicants of patent documents.

Private companies predominate the sector (Figure 3), with 38.87% of the deposits of patent documents, followed by independent inventors (34.58%), universities (14.75%), and government institutions (11.80%). It is possible to note that the sum of patent deposits by universities and governmental institutions does not exceed the deposits of the private sector. Then, the need to increase the interactions between universities and the industrial sector is emphasized. The establishment of this partnership can generate financing and exchange of knowledge, in addition to stimulating the process of technology transfer in a cooperative system and the commercial use of the results achieved [54,55]. To this end, the training of professors, and researchers about the protection of intellectual property, its applications in the market, and the stimulus of communication/cooperation with companies through internship programs and Research and Development actions, are essential to result in a greater insertion of universities on patent filing [56]. Another relevant point is the establishment of technology transfer offices, designed by universities which can disseminate research, commercialize inventions for revenue generation, and facilitate interrelationships with other agents of the innovation system (industries and government) [57].

**Figure 4:**
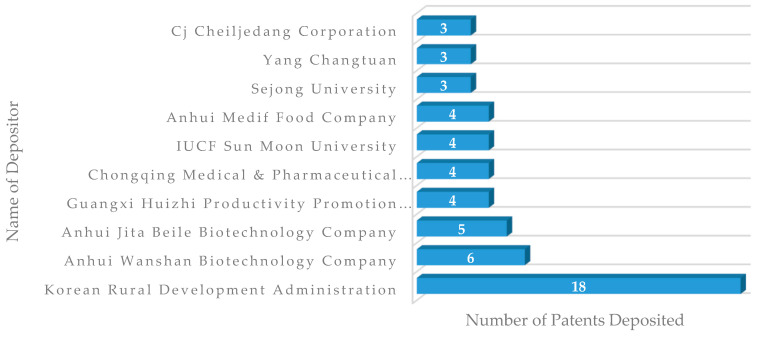
Main depositors of patent documents associated with the development of food products with edible insects.

The Korean Rural Development Administration (RDA) holds 18 out of 341 documents from patents assessed (Figure 4). The RDA is a government institution based in Jeonju City—Republic of Korea. The company is known for fostering research with edible insect species, relevant genetics research, and material extraction, among other topics. Since its establishment in 1962, it has been playing a crucial role in the development of agricultural technologies in South Korea [58].

Most of its patents (15) relate to food compositions with functional/health claims: Antithrombotic (4—KR102150121B1, KR101828294B1, KR101962008B1, KR101758718B1 [59,60,61,62]), anti-inflammatory (3—KR102169046B1, KR101424125B1, KR101382400B1 [63,64,65]), anti-obesity (3—KR102159019B1, KR20180075888A, KR101491771B1 [66,67,68]), antioxidant (1—KR102150122B1 [69]), sexual function improvement (1—KR100462166B1 [70]), internal organ protection (1—KR102121807B1 [71]), hangover improvement (1—KR101055252B1 [72]), and hepatoprotective (1—KR101702053B1 [73]). The document KR101758718B1 [62] has as an object of the invention of a food with the claim of antithrombotic effect, developed with compounds isolated from *Protaetia brevitarsis*. These compounds were identified [74] to be indolic alkaloids with the potential to inhibit platelet aggregation.

Hwang Jae Sam, Kim Mi Ae, and Yun Eun Young were the most frequent inventors and coinventors in RDA filings, with 9, 8, and 9 documents each, respectively. Moreover, these inventors developed scientific studies in recent years that evaluated insect bioactive compounds with beneficial functions for the body, such as glycosaminoglycan derived from *Gryllus bimaculatus*, a substance with potential adjuvant for the treatment of chronic arthritis [75]; and ethanolic extract of *Tenebrio molitor* larvae with anti-obesity effect [76], which is in line with technologies patented by RDA in the area of edible insects.

The Chinese company Anhui Wanshan Biotechnology, located in the city of Hefei, submitted six patent applications regarding insects and food products. All the patents associated with the company report the production of enriched bee larval protein powder with minerals. For example, patent CN103622038A [77] discloses a multifunctional bee larvae protein powder rich in zinc. The high content of the mineral, according to the patent, would be associated with the dietary modulation of bee larvae. Some studies have shown that the nutritional composition of edible insects can be modified through their diet and can even make them sources of nutrients that they commonly would not be if they consumed a natural diet [78,79,80]. Regarding mineral composition, differences are attributed almost exclusively to diet, as minerals are not synthesized in the animal body, but rather accumulated after dietary intake [78]. Moreover, bee protein seems to present potential from a nutritional and environmental point of view and can serve as a substitute ingredient in preparations, such as hamburgers [81], with a balanced amino acid composition [82].

Chongqing Medical and Pharmaceutical College was one of the leading universities to file patents associated with the subject of this study, with a total of four filings. It is a university established in 1948, located in the university town of Shapingba, China, a cultural district in Chongqing. Its structure is composed of 19 colleges, including the Food Nutrition and Testing College [83]. The documents deposited by this University are associated with the application of dragonfly nymphs in foods, such as sausage—CN108077797A [84], canned food—CN108077805A [85], and dumplings—CN108142798A [86] and CN108175042A [87]. According to the documents, dragonfly nymphs have a unique and pleasant flavor, are widely accepted by consumers and their application represents a way to enhance the country’s food culture. In China, the consumption of dragonflies is relatively common in preparations, such as vegetable soup, scrambled eggs, or even roasted dragonflies [88].

## 6. Key Inventors

**Figure 5:**
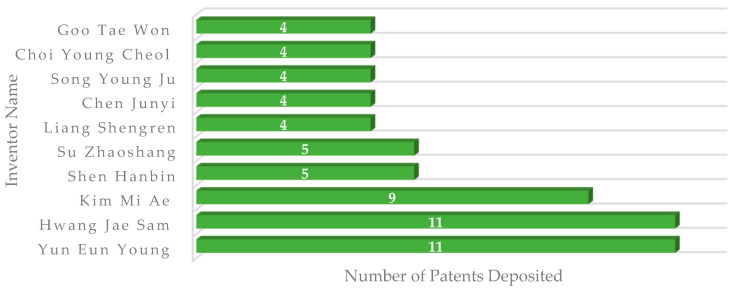
Main inventors of patent documents associated with the development of food products with edible insects.

With regard to the applicants, there is a prevalence of inventors of Asian origin, with the top 10 having at least co-ownership in four patents filed (Figure 5).

The Korean inventor, Eun Young Yun, presented the largest number of patents (11), most of which were filed on behalf of RDA or through partnerships between the RDA and research institutions, such as Sejong University (Seoul, South Korea), where he teaches and researches with edible insects in the Department of Integrative Bioindustrial Engineering [51]. Most of the patented technologies deal with foods with functional and health potential (9). As an example, patent KR101702053B1 [73] refers to the development of a food ingredient consisting of *Allomyrina dichotoma* (beetle) powder. As possible uses, the patent states that the powder can be inserted in meats, sausages, bread, chocolates, sweets, confectionery products, pizza, noodles, gums, ice cream, and dairy products, resulting in foods with hepatoprotective and anticancer potential. In a study developed by Lee et al. [89], in which the inventor Eun Young Yun participated as a co-author, *Allomyrina dichotoma* powder was able to reduce hepatotoxicity in rats, considering the acute and chronic signs. In addition, the fractionation of *Allomyrina dichotoma* extract with ethyl acetate showed cytotoxicity to various tumor cells through the induction of apoptosis and necrosis [89].

Jae Sam Hwang also submitted 11 stored documents, all of them had the RDA as the applicant, possibly since he develops his activities as a researcher at the National Institute of Agricultural Science (NAAS), an institute inserted in the RDA. Most of his patents (9) presented cooperation with researcher Eun Young Yun in the invention process, indicating the presence of a partnership between the university and government agency. As an example of technology produced, patent KR101424125B1 [64] presents *Tenebrio molitor* as a food ingredient in powder or suspension form to be used in the preparation of gums, teas, vitamin complexes, functional foods, tablets, capsules, or beverages in order to confer the antioxidant and anti-inflammatory capacity to the product. In a study developed by the same inventor [90], the extract of *Tenebrio molitor* showed DPPH radical capture activity of 81.17% and nitrite capture of 43.69%, confirming the antioxidant potential described in the patent.

## 7. Origin of Technology

**Figure 6:**
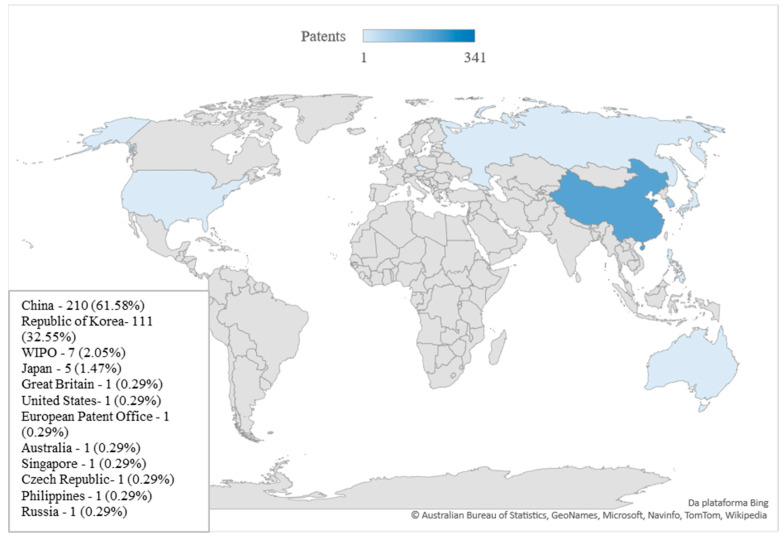
Worldwide distribution of patent documents associated with the development of food products with edible insects.

Of the total patent documents reviewed, China presented 210 deposits, which represents 61.58%, followed by the Republic of Korea with 111 (32.55%), and the remainder distributed among other countries (Figure 6). Together, these countries were responsible for 94.13% of the patent applications related to the use of edible insects in food products. This arrangement is not the same observed for the number of scientific articles related to edible insect technology. According to data evaluated in the Scopus database, although the development of the technology is mainly in China, the US ranks first in terms of scientific publications, demonstrating that China is more focused on developing technologies with market applications.

The entomophagous culture in China has been reported from at least 3000 years ago. In addition, approximately 300 species of edible insects have already been classified in this country [88]. Compared to other countries in the world, China has invested more intensively in green technologies. For example, in 2007, the Chinese government proposed the National Climate Change Program, which focused on controlling greenhouse gas emissions, overhauling agricultural systems, and stimulating research and development [91]. Therefore, between 2000 and 2015, China represented the forefront of technological innovation related to green technologies, with a considerable increase in patents filed in the area [91].

In China, insects are bred for human food, medicine, and animal feed through domestication and partial or full captive breeding [92]. An example, the Chinese company Gooddoctor-Panxi Pharmaceutical is responsible for cultivating *Periplaneta americana* cockroaches for developing medicinal formulations with healing properties [93]. On average, 6 billion cockroaches are raised by the company, annually, which generated a total of USD 684 million in revenue during its period of activity [94]. In 2018, the company Bugsolutely launched the first snack containing silkworm powder in China [95]. It is a snack containing high protein content and uses one of the main byproducts of sericulture: The silkworm after silk collection [96].

The edible insect and insect protein industry in China accounted for USD 49.1 million in 2018. An increasing population adhering to a healthy lifestyle may account for higher market revenue in the future. The economic forecast is expected to increase to USD 115.7 million/year by 2023, which would generate a compound annual growth rate of 18.7% [97].

A large number of Korean scientific and technological productions can be associated with governmental incentives. As an example, in 2006 the “Bio-Vision 2016”, a national investment program in the biotechnology area, was launched with the purpose of making the country one of the largest biotechnology producers in the world. As a result, there was the development of many research institutes acting in conjunction with large companies and the country met a significant stimulus in technological production through patents filed in this area and scientific productions of relevance [98,99].

The Korean government, in 2010, released the Act on Fosterage and Support of the Insect Industry [100], aiming to increase the income of the families of insect breeders, stimulate the national industry, and basic formation for the development of this area. Since then, the production of insects has grown intensively [101]. As an example, Jeju Gold Larva Co., Ltd., a farm established in 2017, is responsible for breeding *Protaetia brevitarsis* beetles. The enterprise developed a product with this insect, which had been patented (KR102056108B1 [102]). It is a formula for hangover control and liver function improvement. The product, called Bengjooya, is for sale on the company’s website [103].

At the Korean market level, the company named Korean Edible Insect Laboratory (KEIL) is a pioneer in the research and development of insect-derived products. The company has been responsible for the large insertion of edible insects in the Republic of Korea in recent years and presents market dominance [104]. KEIL has developed several partnerships with other enterprises, such as Jeongpoong, in the development of products, such as soups and ice cream with insects; CoffeeNie Café, where cookies and energy bars are distributed to approximately 200 stores in the country; and Korea Matsutani Corporation, which prepares confectionery products [104]. The Global Food company has also been developing insect-based products, such as Korean sausage with powdered mealworms [105]. The annual revenue of the edible insect market in South Korea was estimated at USD 16.3 million in 2018. It is projected to increase to USD 32 million by 2023, with a compound annual growth rate of 14.4% [106].

This information corroborates the idea that the edible insect market presents great investment potential. There is still plenty of room for growth, even in countries with more consolidated markets, such as China and South Korea.

## 8. Type of Product Developed

**Figure 7:**
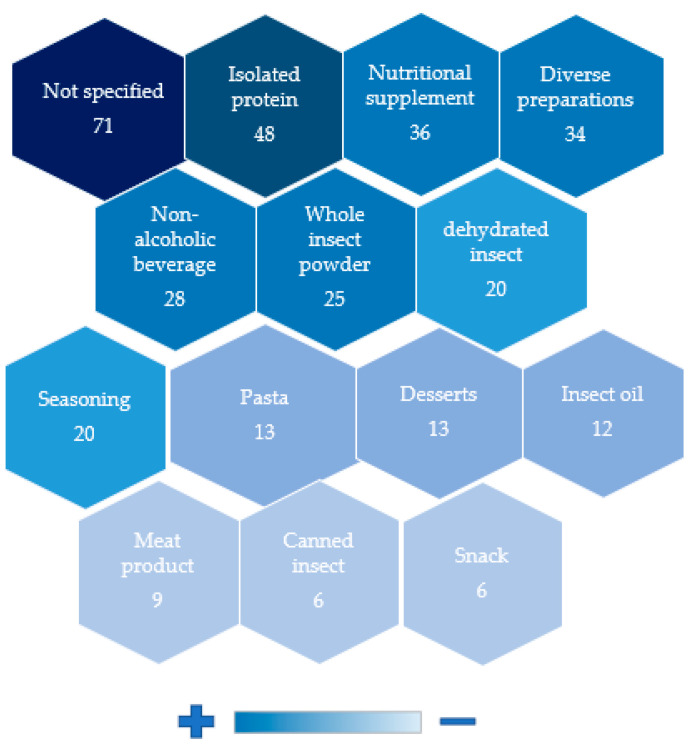
Main types of products developed in patent documents associated with the development of food products with edible insects.

Figure 7 indicates the types of products developed. In general, foods with functional claims were very present in the patent documents. A total of 230 documents dealt with food products with some functional claims. As an example, patent CN107997142A [107] reports a porridge with a constipation improvement claim, containing bee pupa powder. Consumers around the world have become aware of the impact of food on their health and, therefore, have been increasingly consuming functional foods [108]. The global functional food market in 2018 had a total value of USD 161.49 billion, with a forecast to grow to USD 275.77 billion by 2025 [109,110]. Edible insects are important sources of bioactive compounds to be used as ingredients in food products, to generate various health functions, such as antihypertensive, antimicrobial, and antioxidant [111].

Protein isolate powders have been patented 48 times. Patent CN101124936A is intended to provide a method for isolating the protein from *Tenebrio molitor* through refining, filtration, separation, and freeze-drying processes to result in a powdered product. Patent CN109170118A [112] deals with the production of silkworm protein powder. The innovation is the use of a simple extraction process, with high protein yield and little environmental pollution, resulting in a fine, white, odorless powder with high protein content. The protein content of insects is known to be higher than beef or pork [113,114]. Insects can show variation from 40 to 75% protein in dry matter, depending on the species and stage of development [115]. Moreover, insect protein presents excellent quality, as a source of sufficient essential amino acids to meet FAO/WHO requirements for adults [116,117]. In a study developed by Lee et al. [118], the protein digestibility of *Protaetia brevitarsis* larvae was significantly higher than the bovine loin, confirming the feasibility of using insects as a protein source in the food industry.

Protein isolates are acquired through some general technological steps, such as pretreatment, defatting, solubilization, protein recovery and purification, and drying [119]. The resultant can be used as an ingredient in food or as a supplement, to enrich food products, and complement or supplement protein intake in healthy or malnourished people. The use of insect protein isolate can have advantages by masking its odor and other undesirable characteristics, favoring its acceptance as an ingredient in food products; in addition to better absorption by the body through the separation of the protein fraction from components that compromise its digestibility [116,120].

Various preparations were identified in 34 documents. The types of preparations varied from Asian dishes to dumplings. Preparations with rice stand out by their presence in eight patents. Rice is a cereal present in the diet of half of the world’s population. Moreover, approximately 90% of the production and consumption of rice is in Asia, which may justify the findings of this study [121]. For example, patent CN103494094A [122] deals with the development of protein rice containing silkworm and bee powder as ingredients, which according to the document, confer a greater nutritional contribution, in addition to good digestibility. Additionally, patent KR20200004161A [123] presents a method for preparing tofu using an edible insect, citing cricket as one possibility. The patent reports that by adding the insect to the tofu preparation, the protein content can be enriched by approximately 60%.

The high prevalence of products categorized as nutritional supplements in this study (36 times) may be associated with the fact that edible insects have high energy density and high fat, protein, and mineral content [124]. For example, patent CN110150653A [125] deals with the development of a protein bar, containing peanut protein powder, silkworm pupa protein powder, olive oil, modified soy phospholipid, stevioside, konjac flour, glycerin, multivitamin powder, milk, potassium sorbate, and vanilla powder. The ingredients are mixed, formatted as a roll and vacuum packed. Patent KR101622784B1 [126], on the other hand, relates to a diet energy bar containing quinoa, nuts, and the whole powder of an edible insect, thereby taking advantage of the nutritional richness of whole insect use.

Edible insects are already used in several regions of the world as an affordable nutritional supplement due to their quality and higher concentrations of nutrients, despite the perception that they are considered as delicacies or exotic products [124]. Flours and/or insect powder, are excellent options for consumption and incorporation as an ingredient in products since the ingestion of whole insects or parts of them, still causes repulsion in consumers, as they consider entomophagy as a primitive and repugnant practice [127,128]. In the development of vegetable soup enriched with termite (*Macrotermes bellicosus*) based flour, authors observed that the insertion of 23% of the insect flour resulted in an increase in approximately twice the protein content (29.94%) compared to the control soup (15.03%) [26]. Oat biscuits enriched with 5, 10, and 15% *Acheta domesticus* flour showed an increased protein content by 18.35, 36.81, and 55.16%, respectively, compared to the control [129]. The increase in nutritional contribution observed in products with insects occurs since these are important sources of macronutrients, such as proteins, lipids, and carbohydrates; therefore, the use of insects in its processed form (such as flour/powder) can add more nutrients to products and favor the sensory acceptance [31].

Some studies have shown that acceptance of foods containing processed insects is good and tends to vary with their proportion in the products. As an example, bread produced with grasshopper (*Schistocerca gregaria*) flour [23] showed higher sensory acceptance when the grasshopper flour was present at a lower concentration. Muffins developed with cricket powder [130] had wheat flour replacement with 2, 5, and 10% insect flour. Samples with 2 and 5% were found to have higher sensory acceptance with higher taste scores compared to the control. The products with 2, 5, 10%, and the control obtained, respectively, scores of 7.1, 7.0, 6.9, and 6.2 on a hedonic scale of acceptance ranging from 1 to 9. Therefore, it is evidenced that the addition of insects to food products is not harmful to their sensory characteristics. This indicates future trends and the feasibility of food products with insects since from sensory studies it is possible to reach an insect proportion acceptable to consumers, and thus increasing the consumption of a product potentially richer in nutrients.

## 9. Nutrients Associated with Patented Products

**Figure 8:**
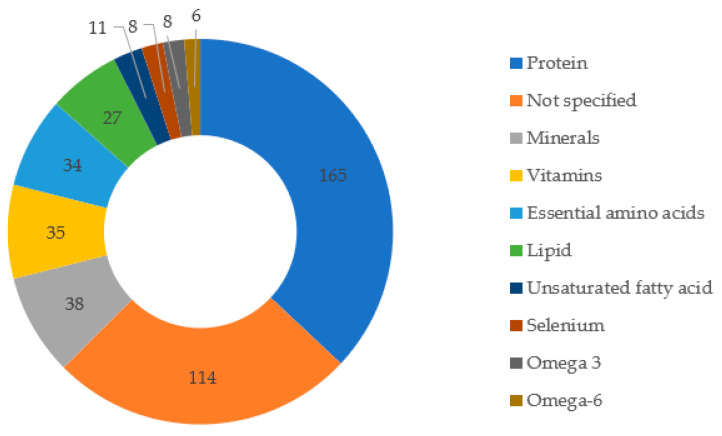
Main nutrients cited as components of the products described in the patent documents.

In relation to the nutrients described in the patent documents (Figure 8), the protein fraction has the highest value, which is cited in 165 documents. The average protein content is variable, ranging from 40 ± 14% for insects of the order *Isoptera* (termites) to 64 ± 20% for insects of the order *Blattodea* (cockroaches) when evaluated on a dry matter basis [131]. Insect protein has good digestibility when compared to casein or soybean; however, it can be improved by removing chitin, the main component of the exoskeleton [132]. Factors that may contribute to variation in the protein content of edible insects of the same species include differences in diet, developmental stage, location, and season of insect collection [131].

Essential amino acids were also among the nutrients in evidence (34 citations). The quality of a protein source is determined primarily by its amino acid composition. Therefore, essential amino acids are key parameters in the evaluation of food quality. According to Akhtar and Isman [132], a large proportion of insects possess a sufficient number of the essential amino acids to meet the daily intake requirements of adults. In a study by Mba et al. [133], all essential amino acids showed a score above 1 in the protein of *Rhynchophorus phoenicis* (beetle) larvae, indicating that they were present in sufficient amounts, considering the protein reference standard for human consumption [117]. The sum of total essential amino acids was two times higher than the WHO reference protein standard and leucine was the most abundant essential amino acid in this insect [117,133]. Ademola et al. [134] found variation in essential amino acid concentration between 44.2 and 46.8% when analyzing the protein quality of *Apis mellifera* (honeybee), *Macrotermes bellicosus* (termite), *Rhynchophorus phoenicis* (beetle), and *Anaphe infracta* (silkworm), which indicates adequacy with WHO [117] requirements for adults. The limiting amino acids in the study were valine in termites and silkworms, as well as threonine in bees and lysine in beetle larvae.

Köhler et al. [15], when analyzing the nutritional composition of grasshopper (*Patanga succincta*), beetle (*Holotrichia* sp.), house cricket (*Acheta domesticus*), and silkworm (*Bombyx mori*), found that only silkworm met FAO/WHO requirements, considering a minimum of 40% essential amino acids and 0.6 essential/non-essential amino acid ratio [135]. In addition, tryptophan was found to be the limiting amino acid in grasshopper and cricket, lysine in beetle, and leucine in silkworm. From these studies, there is great variability between species. However, in general, insects show good protein quality. Moreover, the amino acid gap present in diets rich in cereals, which is usually poor in leucine, lysine, and tryptophan, can be overcome by the consumption of insects [15].

Furthermore, insects are considered lipid sources. According to this study, 27 patents described their products as a lipid source. A study developed by Ray and Gangopadhyay [136] verified total lipid content between 24.16 and 26.21 g/100g dry matter from the indian silkworm (*Samia ricini*). In addition, α-linolenic acid was the most abundant in this species, making up between 38.97 and 40.20% of the total fatty acids. Paul et al. [137] identified a lipid content of 15% in *Acheta domesticus* (cricket) and 32% in *Tenebrio molitor* (mealworm larvae) on a dry basis, with a predominance of α-linolenic acid in *Acheta domesticus* (41.39%) and oleic acid in *Tenebrio molitor* (35.83%). An advantage of insects is the plasticity of their fat composition, which can vary depending on the species and the type of diet [138]. A study developed by Lehtovaara et al. [139] found that the type of diet can significantly modify the fatty acid composition of grasshoppers *Ruspolia differens*, mainly in relation to polyunsaturated fatty acids. Diets rich in linoleic and α- linolenic acids were able to increase 10-fold the content of these fatty acids in the insect.

## 10. Insect Species Used in Patent Documents

**Figure 9:**
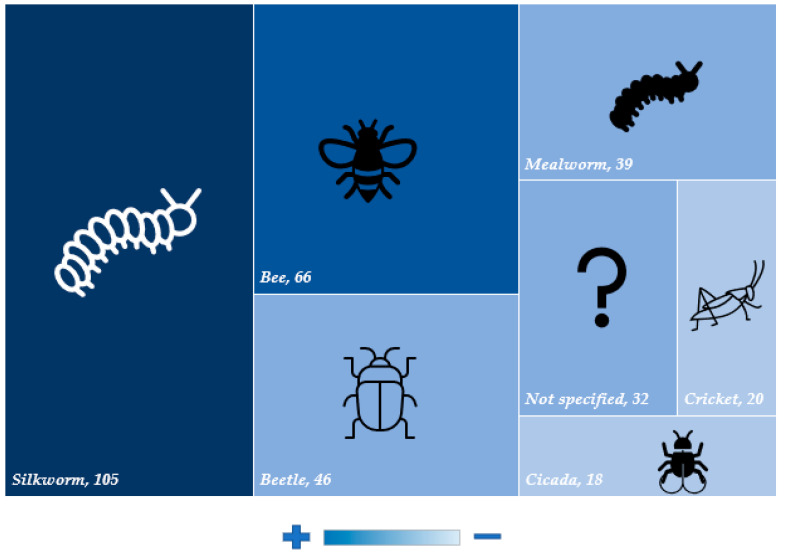
Insects used in the products described in patent documents.

According to Figure 9, the silkworm was the insect most cited in the patent documents (105). The silkworm (*Bombyx mori* L.) has been bred in China for a long time [140], due to the market for silk obtained from its cocoon. Since the beginning of its rearing 5000 years ago, China remains the largest producer of silk and, therefore, the largest producer of silkworms, with an annual production of about 500,000 tons of pupae, accounting for 70% of world production [141].

Silkworm pupa contains about 51% protein and 30% lipids. Moreover, products such as canned and powdered silkworm pupa are available in the Chinese market [141]. In addition, the Chinese Ministry of Health has recently started to consider silkworm pupae as a new source of protein for Chinese consumers, which has generated great interest in research with this insect [141]. Park et al. [142] investigated the physicochemical properties of a meat product developed with the replacement of pork by 5, 10, and 15% of silkworm (*Bombyx mori*) pupae powder. As a result, higher contents of protein were found, from 18.25% in the control to 26.58% with the incorporation of 15% silkworm powder. Akande et al. [143] demonstrated that silkworms can serve as an alternative to meat in the production of pie and pastry fillings for their high protein and mineral content. Furthermore, the amino acid profile showed that the protein is of high quality, highlighting the contents of lysine (8.42%), leucine (7.63%), and total essential amino acids (54.44%). In addition, sensorially, there was no statistical difference between the control sample and the one stuffed with the insect.

Another insect with representation in the documents, was the bee, with 66 patents. Moreover, the bee is an insect of millennial breeding, known for the high nutritional and therapeutic value of the honey produced by it. Similarly, to the silkworm, it is common to consume bee larvae consumption of bee larvae and pupae, especially in Latin America and Thailand [1]. Bees can be considered good sources of protein (35 and 46–57% in larvae and pupae, respectively), and have a lipid profile composed of the fatty acids oleic, linoleic, linolenic, myristic, palmitic, and stearic in large amounts [82]. Ulmer et al. [81] verified the feasibility of burgers by replacing meat with bee biomass. As a main result, it was proposed that to achieve the average protein content of a hamburger (30%), only 462 g of bee biomass would be necessary, a value significantly lower than meat (1 kg), indicating better yield and, consequently, lower environmental impact.

Mealworm was described in patent documents 39 times, and *Tenebrio molitor* was specifically mentioned 18 times. Among the mealworms, three species are most produced at the commercial level: *Tenebrio molitor*, *Zophobas atratus,* and *Alphitobius diaperinus* [144]. A study developed by van Broekhoven et al. [144] verified lipid content between 18.9 and 27.6% of dry matter for *Tenebrio molitor*; 32.8–43.5% for *Zophobas atratus*, and between 13.4 and 24.3% for *Alphitobius diaperinus*, rich in palmitic, oleic, and linoleic acids in the three species. The highest protein content was found for *Alphitobius diaperinus* (65.0%), followed by *Tenebrio molitor* (48.6%), and *Zophobas atratus* (42.5%). Concerning products made with mealworms, García-Segovia et al. [145] developed extruded snacks with the addition of 5% *Alphitobius diaperinus* or *Tenebrio molitor* powder and verified an increase in protein content of 33.57 and 25.73%, respectively. In addition, *Tenebrio molitor* powder was able to significantly increase the selenium content in the snack compared to the control. Breads fortified with 5 and 10% *Tenebrio molitor* were developed and have presented an increase of up to 27% in protein content compared with control bread (wheat flour). In addition, bread fortified with 10% *Tenebrio molitor* showed higher amounts of tyrosine, methionine, isoleucine, and leucine [22]. In 2018, the European Food Safety Authority (EFSA) was invited to provide an opinion on the use of *Tenebrio molitor* in the food industry, in its whole form or as an ingredient in food product development. As a final opinion, the use of *Tenebrio molitor* was considered safe for food application in Europe [36]. In the Republic of Korea, the Korean Food and Drug Administration (KFDA) authorized the use of *Tenebrio molitor* as a safe food ingredient in 2015 [11].

According to the present study, cricket was cited in 20 patent documents. The species *Acheta domesticus* L., known as house cricket, has been used as a food source worldwide [146]. Thailand, in specific, is one of the countries with the largest breeding of crickets in the world, containing, approximately, 20,000 insect factories [147] and even developed the guidelines for the rearing of crickets to standardize production methods and, consequently, maintain a minimum quality [148]. In addition, the Korean Food and Drug Administration (KFDA) authorized the use of *Gryllus bimaculatus*, another known and globally produced cricket species, as a safe and applicable ingredient in the food industry [22]. Kulma et al. [149] found the lipid content for *Acheta domesticus* L. between 12.9 and 21.7 g/100 g dry matter and proteins between 61.2 and 69.6 g/100 g. Udomsil et al. [150] when evaluating the composition of *Acheta domesticus* and *Gryllus bimaculatus*, reported protein contents of 71.7 and 60.7% and lipids of 10.4 and 23.4%, respectively. The predominant fatty acids in both species were palmitic, oleic, stearic, and linoleic. All essential amino acids were present, with higher values of valine, leucine, isoleucine, and lysine. Pasta developed with cricket powder (0, 5, 10, and 15%) as a substitute for semolina, showed increases in lipid concentrations (1.31, 2.45, 3.59, and 4.73% and protein (9.96, 12.27, 14.60, and 16.92) as a function of increasing the concentration of insect powder [151]. Bawa et al. [152] incorporated cricket powder in bread and biscuits and found higher contents of protein, iron, and phosphorus. In addition, consumer sensory acceptability was found to be comparable to control products.

## 11. Functions Associated with the Consumption of Patented Products

**Figure 10:**
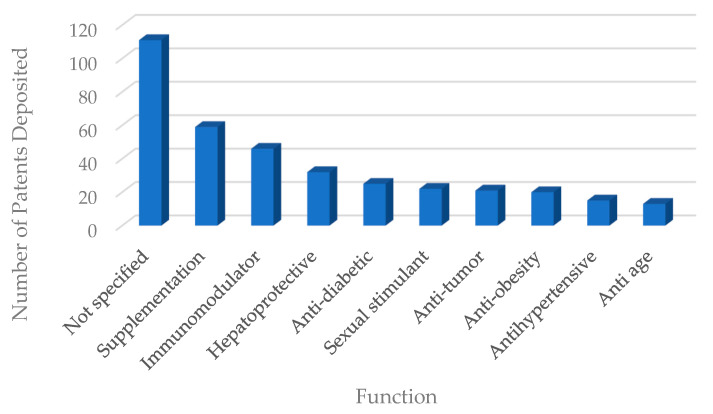
Main functions associated with the consumption of the patented products.

Nutritional supplementation was most frequently cited as a function for the products described in the patent documents (59 citations), according to Figure 10. As an example, patent CN111296833A [153] developed nutritional tablets containing bee extract to supplement athletes.

In general, insects have a strong connection with traditional medicine in several countries around the world. According to Seabrooks and Hu [154], more than one hundred molecules isolated from insects with bioactive potential have been identified in recent years, some of which are described in Table 2.

Immunomodulation was cited 46 times as a function associated with the consumption of insect-containing products. As an example, patent CN105942197A [155] deals with the development of pasta containing insect powder. As a claim, the patent states that the consumption of pasta can strengthen the immune system. Chitin is a component of the exoskeleton of crustaceans and insects that can generate stimulation in cells of the innate immune system [156]. One of the justifications for the function of immune stimulation would be the presence of insect chitin in patented food products.

Ali et al. [157] identified a bioactive polysaccharide (Table 2) in the larvae of the black soldier fly (*Hermetia illucens*) as a molecule that activates the mammalian innate immune response. The molecule, named dipterose-BSF, is capable of stimulating cytokine production in macrophages via the TLR signaling pathway.

The hepatoprotective function was cited 32 times in patent documents. As an example, patent KR101852840B1 [158] describes a formulation containing larvae extract, which, after enzymatical treatment with peptidases, alkalases, and proteases, ensures hepatic protection potential. In a study developed with *Tenebrio molitor* (Table 2), it was observed that alkalase hydrolysate from this insect can protect hepatocytes from cytotoxicity induced by reactive oxygen species, through a mechanism of upregulation of antioxidant genes dependent on nrf-2 [159]. The administration of cricket extract demonstrated that the ability to improve alcohol-induced acute liver injury in mice is possible by controlling oxidative stress [160].

The hypoglycemic function is described in 25 patent documents evaluated. Patent CN105661547A [161] relates to a nutritional supplement containing active probiotic microcapsules, aminobutyric acid, xyllo-oligosaccharide, zinc-rich yeast, mulberry leaf polysaccharide, silkworm pupa peptide, and dietary fiber. The formulation, according to the patent, confers an anti-diabetic effect, by the synergistic action of silkworm peptides with other bioactive substances present. Lee et al. [162] were able to isolate fractions with inhibitory activity of the enzyme α-glucosidase from the E5K6 peptide (Table 2), found in the silkworm cocoon (*Bombyx mori*). The peptide would be useful in reducing postprandial hyperglycemia in patients with diabetes.

Another function cited in the patent documents was anti-tumor (21 documents). The CN101683166A patent [163], for example, deals with the preparation of dehydrated mealworms (*Tenebrio molitor*) ready for consumption. As an advantage, the patent states that the ingestion of dehydrated larvae would improve the body’s metabolism and confer an anticancer effect. Wu et al. [164] found that caspase 3-mediated cytotoxicity in human hepatocellular carcinoma and colorectal adenocarcinoma, caused by an oil extract of mealworm (*Tenebrio molitor*), may corroborate the anticancer function associated with the consumption of this insect. A similar study was able to isolate a new oxazole from the insect *Aspongopus chinensis*, in addition to three known N-acetyldopamine derivatives (Table 2) and verify variable cytotoxicities of these molecules against different types of tumor cells [165].

Anti-inflammatory and antioxidant functions were cited 12 and 11 times, respectively. Patent KR20190050540A [166] deals with the method for preparing formulation containing cricket extract. The formulation can be presented in powder, granule, or liquid form, and, by undergoing the action of proteolytic enzymes, presents high content of free amino acids, in addition to antioxidant and anti-inflammatory potential. When evaluating in vitro protein digestion of *Gryllodes sigillatus*, *Tenebrio molitor* and *Schistocerca gragaria* bioactive peptides were found with anti-radical activity via ABTS and DPPH analyses, in addition to LOX and COX-2 inhibitory activity and Fe chelating capacity2 + [167]. Moreover, chitin and chitosan extracted from *Calliptamus barbarus* and *Oedaleus decorus* presented antioxidant activity and antimicrobial function against pathogenic microorganisms, indicating the potential for use in the food industry [168]. Tang et al. [169] developed an experiment that was able to isolate 13 non-peptide nitrogen compounds from *Polyrhachis dives*. Most of these substances are composed of pyridine moieties and three are alkaloids, which were evaluated for the potential of kidney protection, T and B lymphocyte proliferation, and inhibition of TNF-α, COX-1, COX-2, and Jak3 kinase. It was noticed that part of the molecules presented activity in one or more of these assays, indicating anti-inflammatory potential. Yan et al. [170] were able to isolate four new compounds possessing a 2,3-dihydrobenzo [b] [1,4] dioxin group, together with five N-acetyldopamine dimers from *Blaps japanensis*. All compounds were found to exhibit inhibitory effects for COX-2. Ahn et al. [75] evaluated the possible anti-inflammatory effects of glycosaminoglycan obtained from *Gryllus bimaculatus* and found the molecule useful for the treatment of inflammatory diseases, such as chronic arthritis.

The use of insects for medicinal purposes is described in most medical systems in various regions of the world. The historical record shows that the use of insect-based remedies is an ancient practice, which can be found in ancient Egyptian pharmacological texts, as well as being present in Mesopotamian, Roman, and Greek medical conducts [171]. Other bioactive molecules have been isolated from insects, such as residual polysaccharide extracted from *Periplanata americana* [172] with healing potential through stimulation of collagen deposition, polarization of M2 macrophages, and angiogenesis, new isoflavone present in *Periplaneta americana*, with inhibitory effect against the bacterium *Bacillus subtilis* [173], and indolic alkaloids present in *Protaetia brevitarsis seulensis* with antithrombotic and platelet aggregation inhibition potential [74].

**Table 2:** Molecules isolated from insects and their respective biological functions.

Reference	Insect	Bioactive Molecule	Function
Cho and lee [159]	*Tenebrio molitor*	Peptides: Ala-Lys-Lys-His-Lys-Glu Leu-Glu	Hepatic protection
Wang et al. [172]	*Periplanata americana*	Residual polysaccharide	Wound healing
Ali et al. [157]	*Hermetia illucens*	Polysaccharide “dipterose-BSF”	Immunomodulator
Zielińska, Baraniak, and karaś [167]	*Gryllodes sigillatus, Tenebrio molitor,* and *Schistocerca gragaria*	Bioactive peptides	Antioxidant and anti-inflammatory
Gao et al. [173]	*Periplaneta americana*	Isoflavone 13,13-dimethyl,12-(16-hydroxy,16,16-dimethyl)-propanol-∆11,12-hydrogenated pyranyl-7,8 [benzo]-4′-methoxyisoflavone	Antibacterial
Lee et al. [74]	*Protaetia brevitarsis* *seulensis*	Indolic alkaloids	Inhibition of platelet aggregation
Kaya et al. [168]	*Calliptamus barbarus* and *Oedaleus decorus*	Chitin and chitosan	Antimicrobial and antioxidant
Tang et al. [169]	*Polyrhachis dives*	13 non-peptide nitrogen compounds	Anti-inflammatory, immunosuppressive, and renoprotective
Yan et al. [170]	*Blaps japanensis*	four new compounds with a 2,3-dihydrobenzo [b] [1,4] dioxin group, together with five known dimers of N-acetyldopamine	Anti-inflammatory
Ahn et al. [75]	*Gryllus bimaculatus*	Glycosaminoglycan	Adjuvant anti-inflammatory in chronic arthritis
Luo et al. [165]	*Aspongopus chinensis*	Oxazole and 3 components of N-acetyldopamine derivatives	Anti-tumour
Lee et al. [162]	*Bombyx mori*	E5K6 peptide	Hypoglycemic

## 12. Concept Clusters Associated with Patent Documents

**Figure 11:**
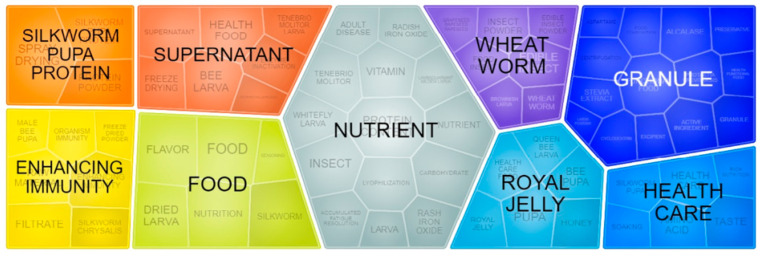
Clusters of concepts related to patent documents involving edible insects and food products. Source: Q. Orbit^®^.

Figure 11 shows how the clusters related to the topic under discussion were organized. It can be seen that the formation of nine clusters, where concepts are present, are more predominantly associated with each other in the patent documents. Two of these clusters are directly associated with specific insects: Silkworm pupa protein and wheat worm. Moreover, these insects are cited in other clusters, in the form of silkworm chrysalis, silkworm, silkworm pupa, *Tenebrio molitor,* and *Tenebrio molitor* larva, which would confirm that they have been more extensively inserted in patent documents and, therefore, more explored. This is in line with the scientific literature since it is reported that these are insect species of great utility for food due to their nutritional quality, in addition to the high capacity of breeding on an industrial scale due to the ease of handling [47].

For the silkworm, there is a noticeable emphasis on the use of its protein, especially in powder form, and an association between its pupa and the preparation of products with health benefits and the application of the spray-drying process. Methods of isolation and application of silkworm peptides in food have been patented due to their various functional effects in the human organism, such as modulation of immunity (CN105661554A [174]); antihypertensive (CN107779489A [175]); and hypolipemiant (CN113647637A [176]). The use of spray-drying may be in evidence since there are reports in the literature of its ability to help improve the OH^−^ radical sequestering capacity and the reducing power of silkworm protein hydrolysates, in addition to low cost and higher production yield compared to freeze-drying [177].

With regard to the silkworm, the cluster of the same name presents concepts, such as insect powder, edible insect powder, edible insect, and food insect, which may indicate its use in a comprehensive way in order to fortify foods nutritionally. As an example, patent KR20190047180A [178] deals with the insertion of mealworm powder in a mix of grains to create a food product rich in nutrients for use in people with nutritional deficiencies. In addition to these insects, there is a cluster called royal jelly, which would encompass the concepts of queen bee larva, pupa, bee pupa, honey, and health care food.

The presence of this cluster can be justified by the fact that most patent documents involving bees deal with the combined use of the various products derived from the breeding of this insect, such as royal jelly, pupae, and honey, in the development of foods with enhanced nutritional and functional benefits, such as jams (CN100998382A [179]), creams (CN102334626A [180]), and jellies (CN112089038A [181]).

The cluster called granule is mainly associated with the term functional food. The granulation method is capable of uniting various powdered substances into a single entity or granule. This can occur to prevent the separation process of certain ingredients in the pharmaceutical and food industry in the development of new products [182]. As an example, patent document KR20200019806A [183] deals with the production of *Protaetia brevitarsis* granules with antidiabetic potential to be used in food products, comprising 55 to 65% by weight of *Protaetia brevitarsis* larval powder, 25 to 35% by weight of lotus root powder, and 5 to 15% by weight of red ginseng extract.

The presence of the clusters enhancing immunity, nutrient, and health care confirm the findings described in this study, where one can see the great potential of insect application in food fortification and in conferring functional power and improvement of human health.

## 13. Economic Projections for the Edible Insects Market

**Figure 12:**
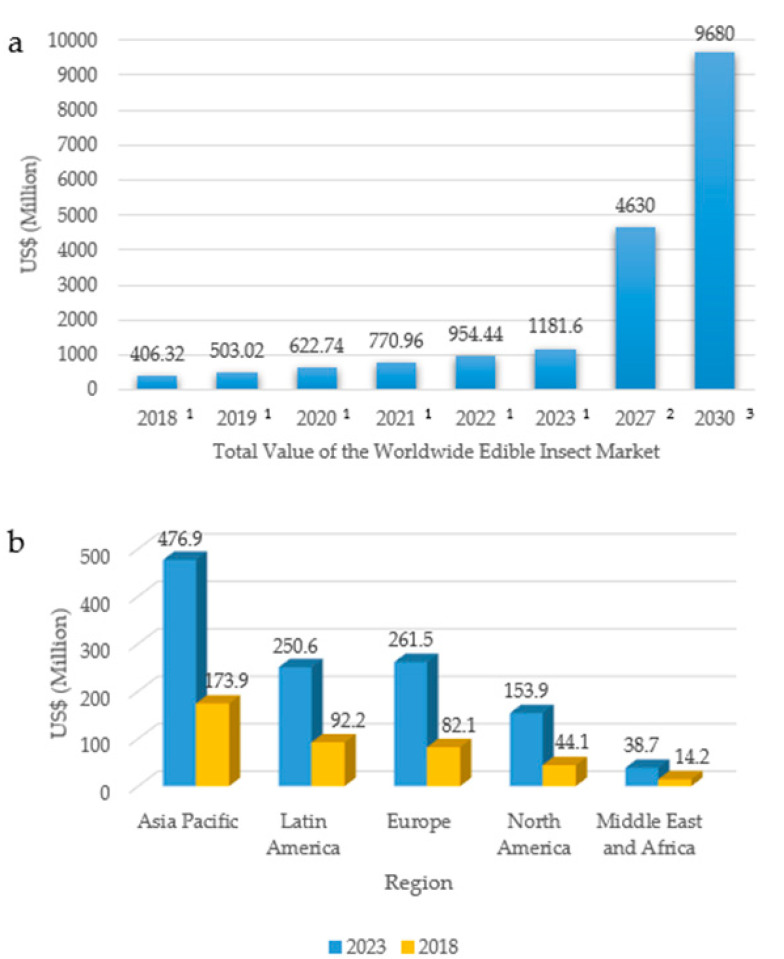
(**a**) Projection for the world market value of edible insects. Sources: ^1^ [184]; ^2^ [185]; ^3^ [186]. (**b**) value of the world market for edible insects by region. Source: [188]. Values presented in USD million.

The number of producers and consumers of insects and their products presents the perspective of an increase in the next years, and the forecast is that, by 2023, the market of edible insects will reach, approximately, USD 1.181 billion (Figure 12a), with a growth of, approximately, 190.80%, in comparison with 2018 (USD 406.32 million) [184]. Recent data [185,186] indicate that this market will continue to grow significantly in the coming years, potentially reaching USD 4.630 billion in 2027 and almost USD 10 billion in 2030. Government and scientific stimuli have resulted in impacts not only in the number of patent deposits or publication of articles in the area, but also in investment in the market. According to Pippinato et al. [187], to satisfy emerging trends worldwide, non-traditional sources of protein, such as insects, are gaining increasing importance.

For 2023, the Asia-Pacific region will maintain a larger market size, growing from USD 173.9 million in 2018 (Figure 12b) to USD 476.9 million by 2023. However, North America stands out as the region with the highest forecast growth in the edible insects market, with an expected compound annual growth rate of 28%, followed by Europe at 26%, Middle East and Africa, Latin America, and Asia-Pacific at 22% [188]. Countries, such as Canada and United States are in the process of the very recent development in edible insects. According to Wilkie [189], until 2012, there were no reports of insect farms for human consumption in North America; however, from 2012 to 2018, approximately 18 farms were built to meet the demand of this new food sector. Apart from this, the North American edible insect market is forecast to increase from USD 44.1 million in 2018 to USD 153.9 million by 2023, which is a total growth of 248.97% [188]. In Europe, regulation of the use of edible insects has created a stimulus for this market, which is forecast to grow from USD 82.1 million in 2018 to USD 261.5 million by 2023 [186]. According to Pippinato et al. [187], the inclusion of insects and their derivatives in the European food industry can be foreseen in the coming years, mainly in the development of protein-source products, to ensure greater diversity in the market.

Entomophagy is quite present in Latin America, where countries such as Brazil, Ecuador Peru, Colombia, and Venezuela stand out in entomophagic habits, due to cultural issues [190]. The edible insects’ market in Latin America, in 2018, presented the second largest economic value, at USD 92.2 million, with a forecast to increase to USD 250.6 million by 2023, which is surpassed by Asia-Pacific and Europe [188]. According to Bermúdez-Serrano [191], there is still a shortage of start-ups in Latin America, and this may be associated with the absence of regulations focused on insect production and commercialization; and strategies specifically created to stimulate the edible insect industry. Therefore, government efforts are still needed to change the view of insects as contaminants in food to make them a new potential for economic development in this region.

According to Baiano [192], the rearing of edible insects is of relevance to making alternative food sources available to the market, considering the trend of population increase. Developing countries in regions, such as Southern and Central Africa, could especially benefit from this. In Africa, however, it is reported that there is a need to improve the organization of food market chains, which would stimulate agribusiness to diversify its production and insert edible insects as a possibility of income generation [193]. Therefore, there are two important issues to be considered to stimulate the market of edible insects. The first is the legal aspect, i.e., it is necessary that countries begin to regulate the production and marketing of insects. The other issue is the need to change the mentality of the Western population that tends to react negatively to the possibility of insect consumption [192].

## 14. Conclusions

The present research scrutinized patent documents related to edible insects in food products while discussing novel scientific articles from a similar subject by a hybrid review method. Therefore, the article contributes to the global mapping of technological and scientific production associated with food products developed with insects. As a result, it is possible to verify the hotspots (i.e., fields in which patents have intensively appeared) as well as technological gaps (i.e., fields in which patents have not yet been granted); therefore, unfolding new opportunities that can be further explored by the market. The information discussed in the present study can help with reducing uncertainties by assisting the consolidated private initiative and start-ups with prior knowledge, increasing systemic competitiveness, and improving decision-making by R&D managers in this specific field.

From the analysis performed through the information contained in patent documents, it is possible to conclude that the application of edible insects in the food industry shows a tendency to develop in the coming years, suggesting that there is still great room for investment in research and market. FAO possibly has a key role in stimulating the development of technologies associated with the use of insects in food products due to the release of the document “Edible insects: future prospects for food and feed security” in 2013, the same year when the period of exponential growth of patent applications began. Edible insects are mainly used as a form of protein supplementation in food products and the silkworm is still the insect with the highest number of patents on the subject studied, possibly due to its high production in the Asian market for consumption and silk extraction. Considering the country of origin of the patented technology, it is possible to report a concentration in the East, with a predominance of patent deposits by China and the Republic of Korea, countries that have more consolidated innovation systems in the area, through joint actions of government incentives and national projects.

For the Western world, cultural rejection of entomophagy can be considered a barrier to more inclusion of insects as food in a global panorama, but that can be overcome with educational actions, such as sensory studies aligned with universities, industry, and the population, as well as other projects for the popularization of science and technology in this theme. The development of foods containing whole insect powder or isolated protein as an ingredient may be an interesting initiative to reduce the prejudice toward eating it, while the capability of the insects of enhancing the nutritional properties of a plethora of globally consumed foods, such as cookies, pasta, and brownies, must be reinforced.

The lack of legislation regarding insect rearing and its human consumption in some countries also needs to be assessed to provide conditions for the development of insect factories and more sustainable food production in those regions, mainly in developing countries that can benefit the most from this high-quality food source.

Overall, the use of insects in the food industry seems to represent a great opportunity to develop products with high added value, in addition to potentially meeting the demand of countries in a situation of food and nutritional insecurity. Considering the unstable global panorama, the stimulation of edible insect rearing and consumption is of great relevance in order to maintain country sovereignty in those regions that may be most affected by food insecurity from the COVID-19 pandemic and Russia-Ukraine war.

Considering the study limitations, some patent documents did not fully describe their inventions, which could underestimate the parameters evaluated. Moreover, the Espacenet database may not cover all the protected patent documents in the world, in comparison with other databases, which also may be a factor of data underestimation. For future perspectives, the present study may be useful as a starting point to indicate directions toward the development of novel, nutritionally enriched, functional food products with different insect species. It can be pointed out that this subject is still in infant research status in many countries that possess an entomophagy culture. Furthermore, few insect species are intensively studied and applied in foods, considering their diversity worldwide and this must be assessed.

## Figures and Tables

**Table 1:** Scope of prospection of patent documents by keywords and IPC/CPC codes in the Espacenet database.

Insect *	Pupa *	Larva *	Nymph *	A23L33	A23V2002	Results
X						>10.000
	X					3.137
		X				>10.000
			X			898
X				X		341
X					X	359
	X			X		227
	X				X	151
		X		X		215
		X			X	137
			X	X		36
			X		X	19
X	X	X	X	X		751
X	X	X	X		X	621
**X**	**X**	**X**	**X**	**X**	**X**	**1139**

## Data Availability

Not applicable.
